# Recent progress in cancer cell membrane-based nanoparticles for biomedical applications

**DOI:** 10.3762/bjnano.14.24

**Published:** 2023-02-27

**Authors:** Qixiong Lin, Yueyou Peng, Yanyan Wen, Xiaoqiong Li, Donglian Du, Weibin Dai, Wei Tian, Yanfeng Meng

**Affiliations:** 1 The Ninth Clinical Medical School of Shanxi Medical University, Taiyuan, Shanxi 030009, China; 2 Department of MRI, Taiyuan Central Hospital of Shanxi Medical University, Taiyuan, Shanxi 030009, China; 3 School of Public Health, Shanxi Medical University, Taiyuan, Shanxi 030001, China; 4 Department of General Surgery, Shanxi Cardiovascular Hospital, Taiyuan, Shanxi 030024, China

**Keywords:** cancer cell biomimetics, nanoparticles, precision medicine, targeted therapy, theranostic nanomedicine

## Abstract

Immune clearance and insufficient targeting have limited the efficacy of existing therapeutic strategies for cancer. Toxic side effects and individual differences in response to treatment have further limited the benefits of clinical treatment for patients. Biomimetic cancer cell membrane-based nanotechnology has provided a new approach for biomedicine to overcome these obstacles. Biomimetic nanoparticles exhibit various effects (e.g., homotypic targeting, prolonging drug circulation, regulating the immune system, and penetrating biological barriers) after encapsulation by cancer cell membranes. The sensitivity and specificity of diagnostic methods will also be improved by utilizing the properties of cancer cell membranes. In this review, different properties and functions of cancer cell membranes are presented. Utilizing these advantages, nanoparticles can exhibit unique therapeutic capabilities in various types of diseases, such as solid tumors, hematological malignancies, immune system diseases, and cardiovascular diseases. Furthermore, cancer cell membrane-encapsulated nanoparticles show improved effectiveness and efficiency in combination with current diagnostic and therapeutic methods, which will contribute to the development of individualized treatments. This strategy has promising clinical translation prospects, and the associated challenges are discussed.

## Review

### 1 Introduction

Biomimetic nanotechnology, an emerging interdisciplinary field, involves different disciplines, such as nanomaterials science, mechanical engineering, pharmacology, and clinical medicine. Nanoparticle (NP)-based therapeutics are uniquely able to improve drug loading efficiency, control drug release, and protect drug molecules against undesired degradation [1–2]. NPs are widely used in various medical fields, such as cardiovascular diseases, neurological diseases, malignant neoplasm, orthopedic diseases, and immune system diseases, providing a new approach to various treatment problems [3]. These nanoformulations provide advantages over conventional pharmaceutical formulations in terms of safety and efficiency. However, there are still many inadequacies that limit the application of nanoformulations in biomedicine, including limited tumor penetration and insufficient specificity. Furthermore, nanoformulations are often recognized as foreign materials by the reticuloendothelial system (RES) or the mononuclear phagocytosis system (MPS) [4]. The subsequent rapid clearance from blood circulation by the liver and kidneys results in insufficient drug accumulation in the target tissue [5]. In addition, NPs can interact with proteins to form a protein corona, which affects the intended function of the NPs, resulting in changes of biological behavior and loss of function [6–7]. Moreover, the protein corona can accelerate RES/MPS uptake and interfere with the targeting ability of NPs [8].

The biomimetic technique of cell membrane coating, which employs naturally cell-derived membranes, provides a new approach to address NP deficiencies [9]. The encapsulation of NPs with cell membranes can endow the NPs with biomimetic functions and replicate the biological characteristics derived from the original cells, such as the immune evasion of erythrocytes [10] and platelets [11] and the tumor-targeting ability of stem cells [12], immune cells [13], and cancer cell membranes [14]. In vitro and in vivo studies have demonstrated that cell membrane-coated nanoparticles exhibit higher potency, longer retention, and more significant accumulation than bare nanoparticles in the tumor environment (TME) because of immune evasion and cancer targeting abilities [15]. Moreover, biomimetic nanoparticles provide significant advantages regarding biocompatibility, low cytotoxicity, and structural support [16].

With the rapid development of biomimetic nanotechnology different types of derived membranes have been exploited for the development of novel membrane NP-based therapies, such as erythrocytes [10], platelets [11], cancer cells [14], stem cells [12], immune cells [13], central nervous system-derived cells [17], bacterial outer membrane vesicles [18], and extracellular vesicles [19]. Cancer cell membranes exhibit distinctive advantages because of their easy cultivation and their superior homologous targeting and immune evasion [20]. Cancer cell membrane-based biomimetic nanotechnology provides a new methodology and exhibits promising prospects [21]. Compared to the potential threat of living tumor cells to patients, cell membrane coating nanotechnology is safer during usage because of the inactivation of tumor cells and the removal of intracellular components [22]. In treatments of tumors that lack effective targeting, cancer cells achieve precise delivery of bionanoparticles to different types of tumors through homotypic aggregation to homologous tissues [23]. The encapsulated NPs can specifically accumulate in the tumor tissue and reduce early clearance, thereby prolonging the residence time of the drugs they carry and reducing their systemic side effects [24]. Furthermore, cancer cell membranes also show excellent performance in nontumor diseases such as immune system diseases [25] and cardiovascular diseases [26] because of their rich surface functions that yield immunomodulation [22] and biological barrier penetration [27].

**Figure 1:**
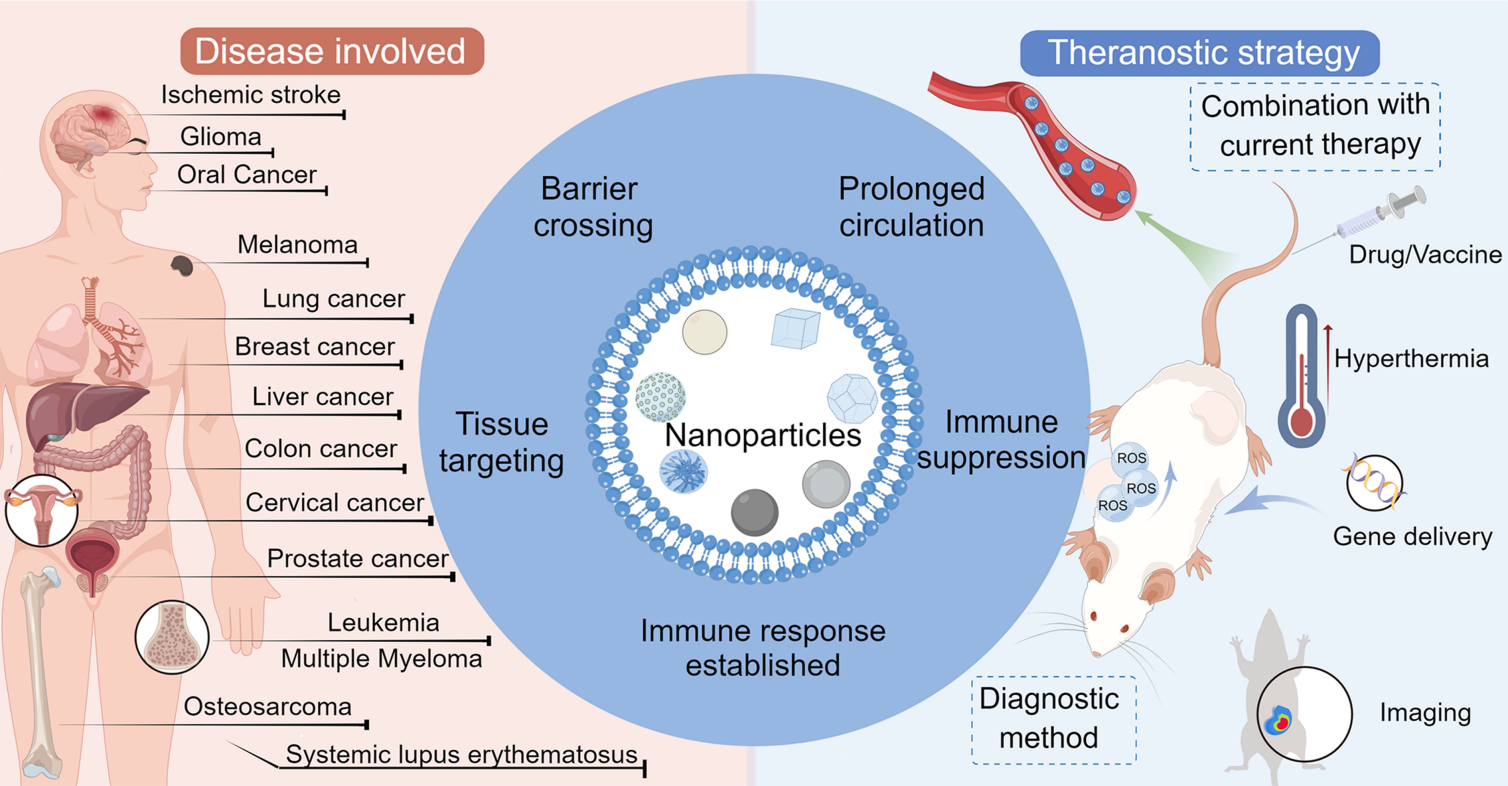
Application of biomimetic cancer cell membrane-coated nanoparticles in different types of diseases: therapeutic and diagnostic methods. Figure 1 was drawn using Figdraw (https://www.figdraw.com), export ID PSWIPb05cf. The materials contained in the image are copyrighted by Home for Researchers. This content is not subject to CC BY 4.0.

Here, we review recent advances and original research in applying biomimetic NPs coated with tumor cell membranes in the medical field and provide a comprehensive summary of the different diseases and diagnostic and therapeutic methods involved (Figure 1). First, different properties of cancer cell membranes are discussed separately. Cancer cells exhibit unique homotypic targeting to tumor tissues, as well as excellent antiphagocytosis, immunomodulation, and biological barrier penetration abilities. Second, the application of cancer cell-based NPs in different types of diseases are discussed (i.e., malignant neoplasms, hematological malignancies, cardiovascular diseases, and immune system diseases). Third, the integration of cancer cell-based NPs in existing therapeutic and diagnostic strategies is presented and discussed, including radiotherapy, chemotherapy, thermotherapy, reactive oxygen species-related therapies, gene delivery, tumor vaccines, and bioimaging of tumors. Finally, the prospects and challenges for the clinical translation of cancer cell membrane-mimetic NPs are discussed.

### 2 The functions of the cancer cell membrane

**Figure 2:**
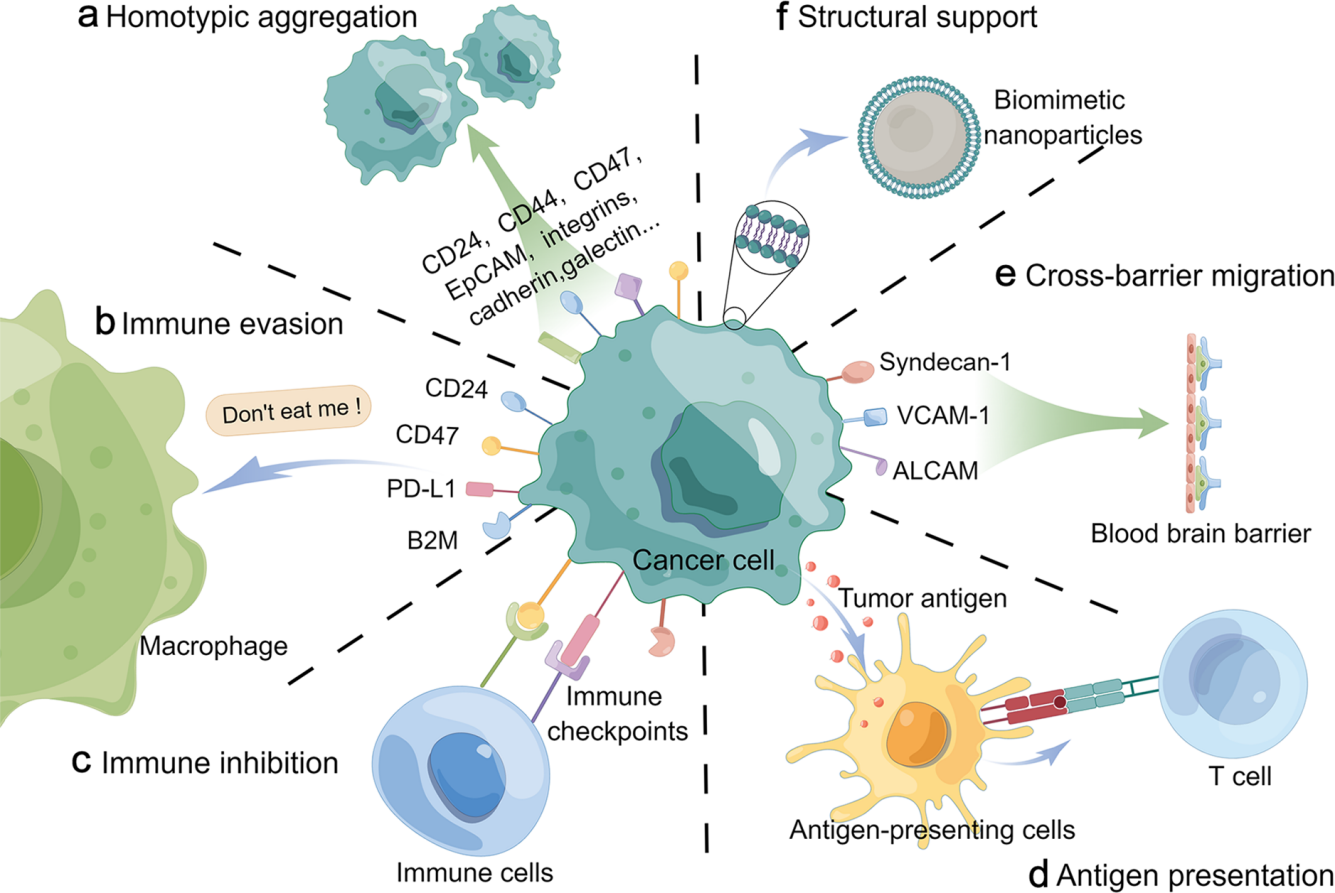
Different roles of the cancer cell membrane. Figure 2 was drawn by Figdraw (https://www.figdraw.com), export ID SWOTY96667. The materials contained in the image are copyrighted by Home for Researchers. This content is not subject to CC BY 4.0.

Different types of proteins present in the cancer cell membrane affect the properties of cancer cells and the way they interact with other cells. The biological effects of nanoformulations can be enhanced through the effective utilization of specific protein groups. A schematic diagram of surface proteins and functions of the cancer cell membrane is shown in Figure 2.

#### 2.1 Homologous targeting

Cancer cells usually exhibit fast growth and easy metastasis [28]. After circulating tumor cells are released from the primary tumor site into the bloodstream, they participate in the main metastasis process and tend to form multicellular homoaggregates at major attachment sites [29–30]. The homotypic aggregation of cancer cells mediates solid tumorigenesis and metastatic behavior [31]. The mechanism of homotypic aggregation is the result of multifactorial action and is closely related to tumor-specific antigens (e.g., Thomsen–Friedenreich antigen [29], carcinoembryonic antigen [32], and glycoprotein 100 [33]) and other adhesion protein ingredients (e.g., EpCAM, N-cadherin, E-cadherin, galectin-1, galectin-3, integrins, CD24, CD44, and CD47 [34–36]) derived from the surface of the cancer cell membrane.

Inspired by these characteristics of cancer cells, biomimetic cancer cell membrane-coated NPs were designed for tumor target delivery. Homotypic cancer cell membrane-modified NPs show stronger targeting capabilities than NPs modified with single ligands [20]. This is attributed to the multiple membrane receptors expressed on cancer cell membranes and their ability to avoid the formation of protein coronas [8,20]. Therefore, cancer cell membrane-encapsulated NPs can achieve better targeting toward tumors.

**Figure 3:**
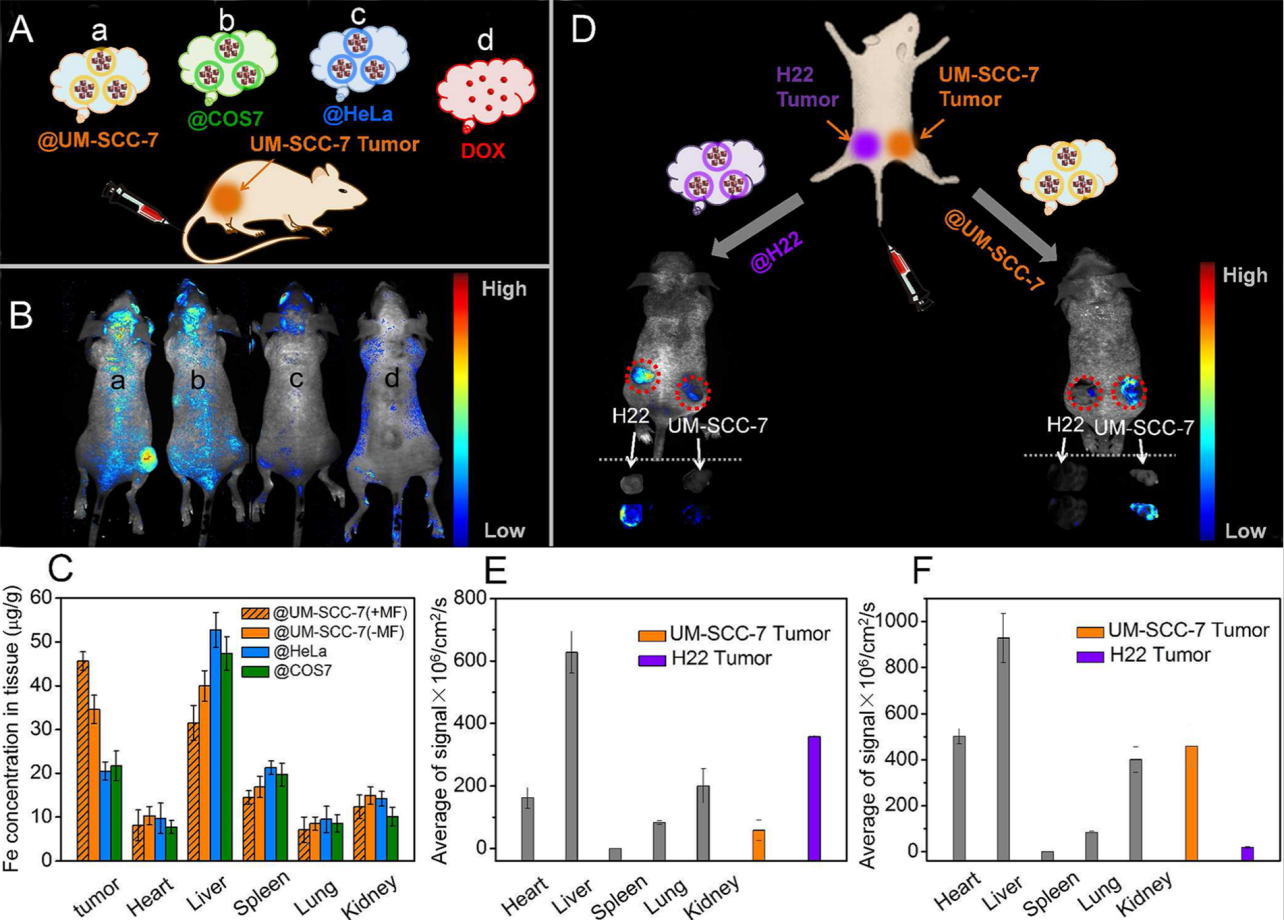
Schematic representation of the distribution of cancer cell membrane-encapsulated NPs in a tumor-bearing mouse model. (A) UM-SCC-7 tumor-bearing mice were injected with Dox (d) and different types of NPs via tail vein (a: UM-SCC-7; b: COS7; c: Hela). (B) Distribution of different NPs with equivalent doses of DOX 24 h after entering UM-SCC-7 tumor-bearing mice. (C) Distribution of iron from NPs in UM-SCC-7 tumor-bearing mice. (D) Biomimetic NPs showed high accumulation only in the same tumor tissue in the dual-type tumor model. (E, F) Fluorescence signal distribution of H22 and UM-SCC-7 membrane-encapsulated NPs. Figure 3 was adapted with permission from [14]. Copyright 2016, American Chemical Society. This content is not subject to CC BY 4.0.

Fang et al. coated poly (lactic-*co*-glycolic acid) (PLGA) NPs with the MDA-MB-435 human breast cancer cell membrane. The encapsulated biomimetic NPs exhibited a stronger affinity for cultured MDA-MB-435 cells in vitro than bare NPs and erythrocyte membrane-encapsulated NPs [37]. Homologous targeting was not found in cells derived from normal tissues. Comparing NPs coated with hepatocellular carcinoma (HCC) HepG2 cell membrane and normal hepatocyte L02 cell membrane, only HepG2 cell membrane-coated NPs showed active recognition and targeted delivery to the tumor [31]. The homologous targeting ability of the cancer cell membrane has been further demonstrated in an experimental murine breast metastasis model. Biomimetic NPs can spontaneously selectively accumulate in primary tumors and metastatic nodules after entering the blood circulation [38]. In contrast to the aggregation to homologous tissues, cancer cells have shown low affinity to other tumor tissues. In the SM-SCC-7 tumor model, biomimetic NPs derived from heterotypic tumor cells did not exhibit homologous targeting to tumor tissue (Figure 3A–C). Furthermore, the biomimetic NPs showed active recognition and homing to the same type of tumor but selectively avoided the heterotypic tumor tissue in a dual-type tumor model (Figure 3D–F) [14]. Additionally, it is interesting that sometimes the cancer cell membrane-coated NPs show a certain affinity for other types of cancer cells, which may be attributed to the expression of cross antigens on different cells. Pan et al. found that breast cancer MDA-MB-231 membrane-coated NPs also showed a certain affinity for the cervical cancer cell line HeLa [39].

#### 2.2 Modulation of the immune system

**Figure 4:**
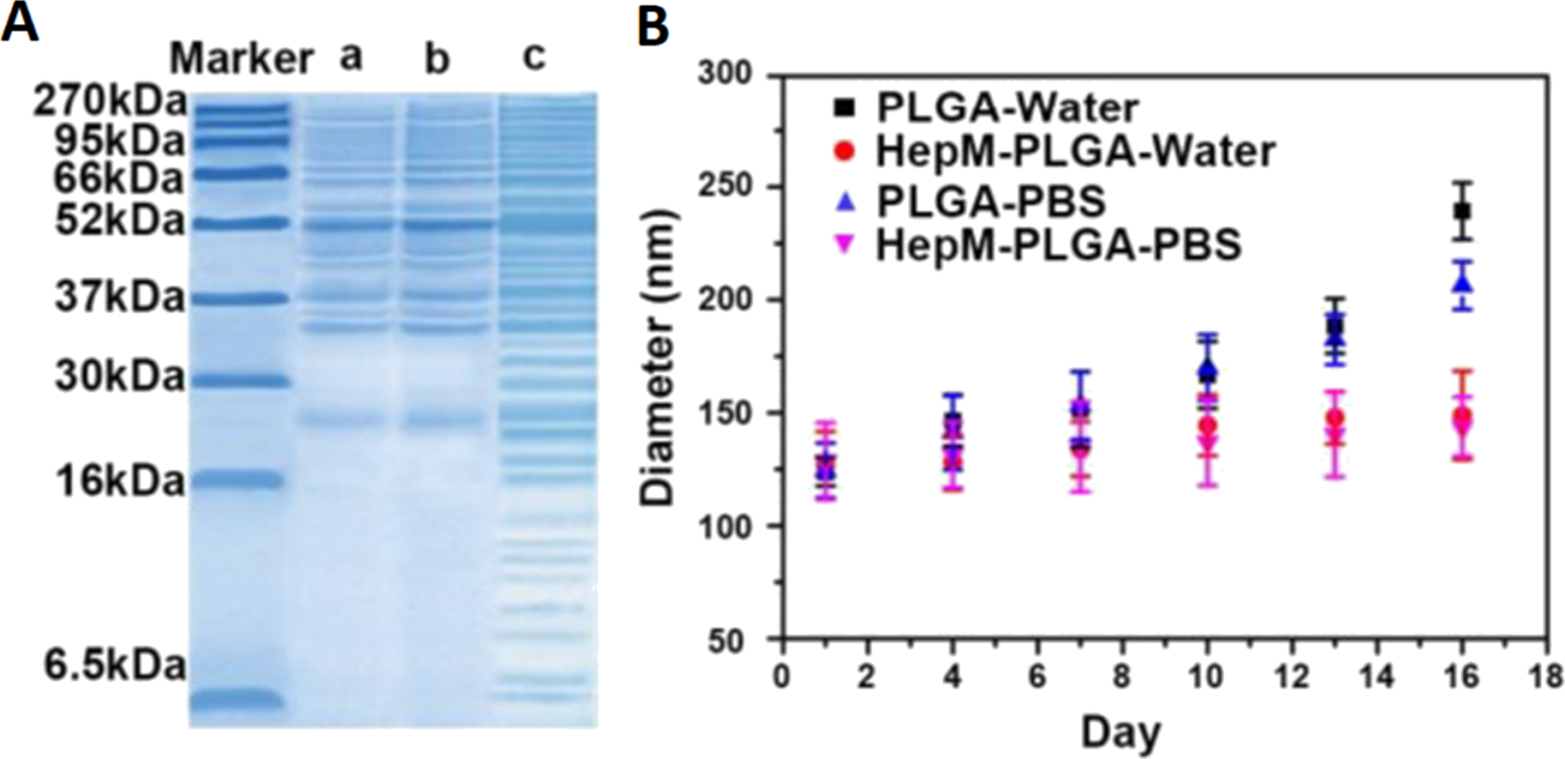
Characterization of membrane-encapsulated NPs. (A) Gel electrophoresis analysis showed that liver cancer biomimetic nanoparticles (a) basically retained and transferred the extracted cancer cell membrane protein components (b), which were similar to the cell lysate (c). (B) The box plot shows that the dimensional stability of cancer cell membrane biomimetic nanoparticles in water and PBS is significantly higher than that of bare nanoparticles. Adapted from [31]. (© 2019 Liu X et al., published by Ivyspring International Publisher, distributed under the terms of the Creative Commons Attribution 4.0 International License, https://creativecommons.org/licenses/by/4.0/).

Cancer cells exhibit excellent immune surveillance evasion, which allows them to continue to expand and form tumors without being cleared from the organism [40]. The immune evasion of cancer cells is closely related to the proteins present on the cell surface, such as CD47, which is ubiquitously expressed on the membrane of solid tumor cells and sends a “do not eat me” signal to phagocytic cells [41]. CD47 has been shown to avoid uptake by macrophages, which enables the NPs to escape immunogenic clearance [42]. Additionally, CD24 has been found to be overexpressed in several malignant diseases (e.g., ovarian and breast cancer). It also counteracts immune clearance by interacting with Ig-like lectin 10 (Siglec-10) expressed by macrophages [43]. Moreover, PD-L1 and B2M play an important role in preventing macrophage phagocytosis [43]. Most of the protein components can be efficiently retained and transferred to NPs during the encapsulation of cancer cell membranes (Figure 4A) [31]. With these features, although NPs encapsulated by a cancer cell membrane are foreign substances, they can still escape the surveillance of the body, thereby resisting phagocytosis by macrophages and prolonging the blood circulation time [20].

The intricate membrane protein components of cancer cell membranes play an important role in immune regulation. Researchers have found a number of immune checkpoint signals in cancers that could confer strong immunosuppression on cancer cells by combining ligands with inhibitory immunoreceptors, such as PD-1, TIGIT, CTLA-4, BTLA, LAG3, and TIM3 [44]. Under reasonable utilization, the immunosuppressive property of tumor cells is expected to be applicable to the treatment of autoimmune diseases [22]. Also, numerous types of tumor antigens that are overexpressed on the surface of cancer cell membranes, after being presented to antigen-presenting cells (APCs), will promote the proliferation and infiltration of active T cells in the TME and induce an antigen-specific antitumor response [33,45]. This natural advantage also makes cancer cell membranes useful in nanoimmunotherapy, which can induce specific antitumor immunity through relevant antigens on the surface of biomimetic NPs [46]. Moreover, the tumor antigens associated with biomimetic NPs show promise in inducing long-term immunological memory, which could prevent cancer recurrence [47].

#### 2.3 Other functions

In addition to the above functions, cancer cells have also been found to have the ability to penetrate the blood‒brain barrier (BBB) in some special cases [26–27]. As a highly specialized structure, the BBB maintains homeostasis of the central nervous system [48]. The targeted delivery of drugs to the brain is challenging because of the limited BBB permeability, which restricts the treatment of brain-related diseases [49]. Aggressive metastases to brain tumors are common in various types of tumors, such as melanoma, lung cancer, and breast cancer [27]. These tumor cells are able to cross the BBB and adhere to brain tissue. This process is closely associated with membrane-associated components such as syndecan-1 [50], vascular cell adhesion molecule-1 (VCAM-1), and activated leukocyte cellular adhesion molecule (ALCAM) [51]. In a study on the ability of cancer cell membranes to penetrate the BBB, NPs coated with cell membranes from melanoma B16F10 cells, breast cancer 4T1 cells, and African green monkey kidney fibroblast COS-7 cells were used to compare the penetration in brain tissue. The NPs coated with B16F10 and 4T1 cell membranes showed excellent fluorescence aggregation signals 8 h after the nanoformulations were administered intravenously, while COS-7 cell membrane-coated and bare NPs showed no obvious signal aggregation [52]. Hence, specifically prepared biomimetic NPs may enable the diagnosis and treatment of brain-related diseases. Additionally, the membrane-encapsulated NPs were shown to have better dispersion and longer storage time than bare NPs because of the higher colloidal stability of the prepared colloidal particles [53]. In one study, liver cancer cell membrane-encapsulated PLGA NPs maintained a stable size in water and PBS for a long time, while bare PLGA NPs progressively grew larger (Figure 4B) [31].

### 3 Application of cancer cell membrane-encapsulated NPs

Regarding current therapies, patients respond differently to the same treatment because of different degrees of disease progression and individual differences and inevitably suffer from a certain degree of toxicity and side effects. Therefore, it is necessary to customize treatments to maximize the benefit of the treatment for the patient at the lowest cost. Cancer cell membranes hold great promise for personalized precision therapy because of their unique homologous targeting properties and immune evasion capabilities. Cancer cell membrane-based biomimetic NPs have been widely used in the diagnosis and treatment of tumor-related diseases (e.g., head and neck tumors [53], gland tumors [9], respiratory system tumors [54], digestive system tumors [55], urinary system tumors [56], gynecological tumors [57], skeletal system tumors [58], and hematological malignancies [59]). Additionally, due to the abundantly expressed functional components, they can play a unique role in specific types of noncancerous diseases, such as cerebrovascular diseases [26] and immune system diseases [22]. Different types of diseases associated with cancer cell membranes are summarized in Table 1 and described in detail in the following sections.

**Table 1:** Applications of biomimetic cancer cell membrane-coated NPs.

Types of diseases		Cell membrane	Core nanoparticle	Drug	In vivo animal model	Ref.

malignant neoplasms	brain tumors	B16F10, 4T1	poly(caprolactone) and pluronic copolymer F68	indocyanine green	BALB/c nude mice	[52]
	breast cancer	4T1	lanthanide-doped upconversion NPs	doxorubicin	female BALB/c nude mice	[23]
	liver cancer	HepG2	PLGA^a^	doxorubicin	BALB/c nude mice	[31]
	prostate cancer	RM-1	mesoporous polydopamine NPs	chloroquine	male BALB/c nude mice	[56]
	cervical cancer	Hela, RBCs^b^	mesoporous prussian blue NPs	indomethacin, gamabufotalin	female BALB/c mice	[57]
	osteosarcoma	143B, RAW264.7	PLGA	paclitaxel	male BALB/c nude mice.	[58]
mematological malignancies	leukemia	C1498	PLGA	CpG oligodeoxynucleotide 1826	C57BL/6 mice	[59]
	multiple myeloma	ARD	PCEC^c^ NPs	bortezomib	C57BL/KA mice	[60]
cardiovascular disease	ischemic stroke	4T1	pH-sensitive polymeric NPs	succinobucol	tMCAO rats^d^	[26]
immune system diseases	SLE^e^	MHCI^f^-deficient 4T1	DSPE-PEG_2K_^g^	dexamethasone	MRL/lpr mice	[22]

^a^PLGA: poly(lactic-*co*-glycolic acid); ^b^RBCs: red blood cells; ^c^PCEC: poly(ε-caprolactone)-PEG-poly(ε-caprolactone); ^d^tMCAO: transient middle cerebral artery occlusion; ^e^SLE: systemic lupus erythematosus; ^f^MHCI: major histocompatibility complex class I; ^g^DSPE-PEG_2K_: 1,2-distearoyl-*sn*-glycero-3-phosphoethanolamine-*N*-[methoxy(polyethylene glycol)].

#### 3.1 Malignant neoplasms

**Figure 5:**
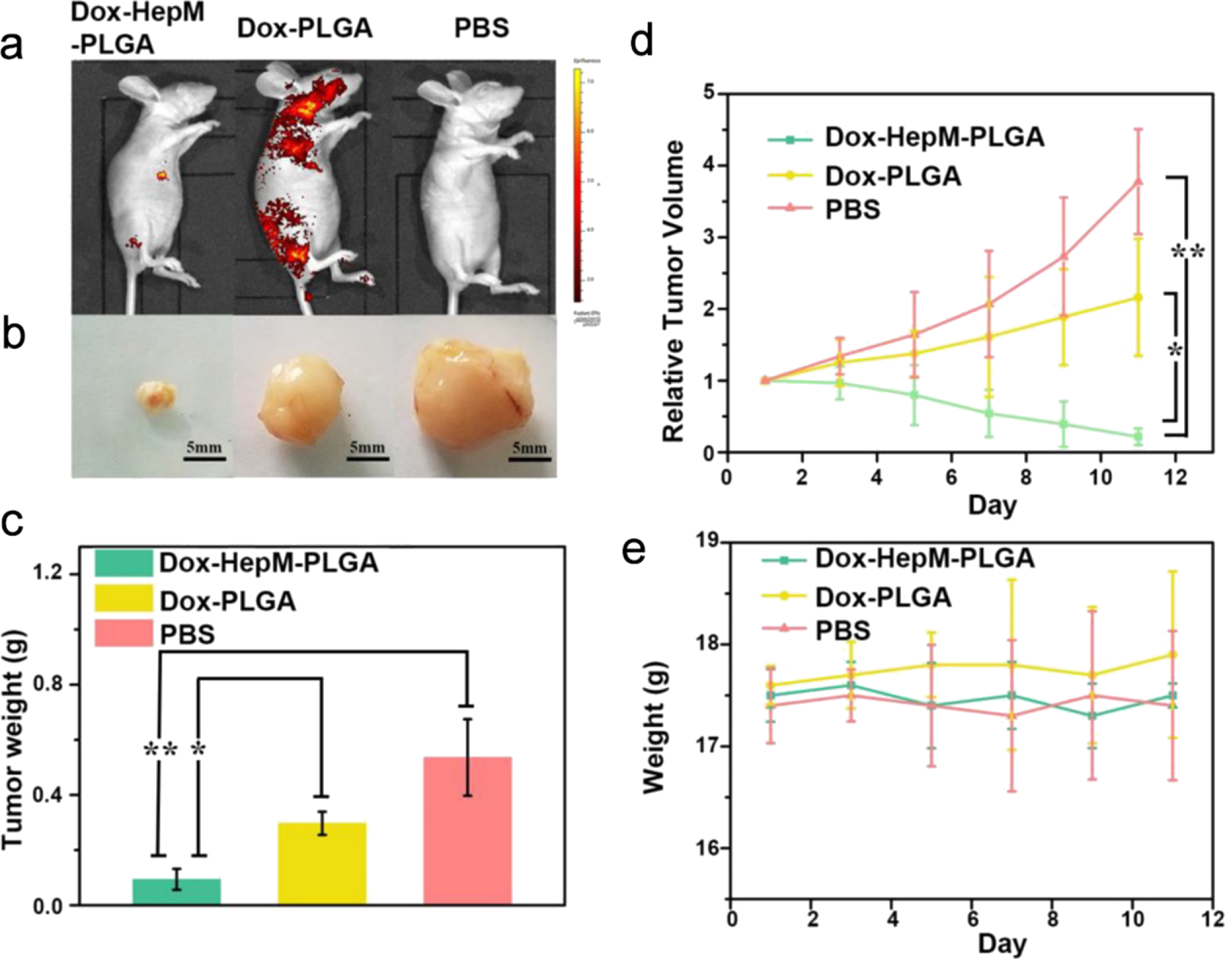
Antitumor efficacy of cancer cell membrane-coated NPs in a hepatocarcinoma mouse model. Hepatoma HepG2 cell membrane-encapsulated PLGA nanospheres loaded with doxorubicin (Dox-HepM-PLGA) yielded smaller tumor volumes than the bare nanoparticles and the PBS control group. (a) Fluorescence imaging eleven days after intravenous injection of biomimetic nanoformulations. (b) Tumor volume. (c) Tumor weight. (d) Relative tumor volume. (e) Changes in mouse body weight. Adapted from [31]. (© 2019 Liu X et al., published by Ivyspring International Publisher, distributed under the terms of the Creative Commons Attribution 4.0 International License, https://creativecommons.org/licenses/by/4.0/).

Insufficient targeting of tumor tissue has hindered patients from further benefiting from therapy. Exploiting the homotypic aggregation behavior during initiation and progression of solid tumors [31], biomimetic cancer cell membrane-coated NPs have shown great promise. This strategy could enable the drug to accumulate in target tissues while reducing accumulation in off-target areas. HCC, a common digestive system tumor, presents the sixth leading cancer incidence and the third leading mortality worldwide [61]. A biomimetic particle model using PLGA NPs as a carrier for the anticancer drug doxorubicin (Dox) encapsulated by the HepG2 liver cancer cell membrane was designed for the treatment of HCC [31]. The HepG2 cell membrane-encapsulated NPs exhibited superior antitumor effects compared to bare NPs and PBS controls under the same treatment conditions in a mouse HCC model. Through homotypic aggregation and immune evasion, as well as the prevention of premature drug leakage from the biomimetic NPs, the drug was efficiently accumulated at the target site. The fluorescence signal showed a predominant accumulation of HepG2 cell membrane-coated NPs in the tumor region after eleven days of intervention, whereas such accumulation was not observed in the bare NP- and PBS-treated groups (Figure 5a). The tumor volume and weight decreased by approximately 90% after eleven days of intervention, while the tumors treated with the bare NPs and PBS showed an increasing growth trend (Figure 5b–d). In addition, all mice showed no significant changes in body weight during the intervention period (Figure 5e) [31]. The efficacy in the HCC model demonstrates the critical role of cancer cell membrane-based NPs in targeting tumors.

Biomimetic cancer cell membrane-encapsulated NPs offer new possibilities regarding cancers that lack therapeutic targets. Breast cancer is the most common malignant tumor in women and the leading cause of cancer death in women [61]. Triple-negative breast cancer (TNBC), as a highly aggressive subtype of breast cancer, accounts for approximately 10–20% of all breast cancers [62]. TNBC shows deficient expression of estrogen receptor (ER), progesterone receptor (PR), and human epidermal growth factor receptor 2 (HER2) [62]. The treatment of TNBC mainly relies on chemotherapy and surgical resection. Due to the lack of effective therapeutic targets and its insensitivity and resistance to drugs, TNBC has a high possibility of recurrence and a poor prognosis [63–64]. A type of multifunctional NPs encapsulated with the membrane of 4T1 breast cancer cells was prepared to counteract the insufficient targeting. The 4T1 cell membrane-coated NPs exert multiple antitumor functions after entering the blood circulation and targeting tumor tissues [23]. This targeted strategy, which does not depend on ER, RR, or HER2, shows good prospects for the treatment of metastatic TNBC.

#### 3.2 Hematological malignancies

Malignant tumors originating in the blood and blood-forming systems, such as leukemia, lymphoma, and multiple myeloma (MM), endanger health and life worldwide. Because of the lack of a single location, leukemia cannot be treated with local interventions such as surgical resection. The main therapeutic approaches rely on chemotherapy, and other treatment methods usually cannot to achieve therapeutic effects. Acute myeloid leukemia (AML) is the most common type of leukemia and is characterized by rapid progression and high mortality [65]. Moreover, AML shows a heightened risk of recurrence through minimal residual disease [65–66]. Cancer cell membrane-based NPs have shown unique advantages in the treatment of leukemia. Johnson et al. prepared AML cell membrane-coated NPs with an immunostimulatory adjuvant [59]. The nanoformulation could induce leukemia-associated antigen-specific T-cell responses after the presentation and delivery of leukemia membrane-associated antigens by APCs [59]. These bionanoparticles showed the advantage of multiple antigens and significantly prolonged survival. MM, a malignant proliferation of plasma cells derived from the bone marrow, is the second most common hematological malignancy [67]. Treatment attempts suffer from the inability to effectively target therapeutic drugs into the bone marrow to effectively kill MM cells [60]. MM cell membrane-encapsulated NPs loaded with the first-line treatment drug bortezomib have been designed to address deficiencies in the treatment process [60]. Utilizing membrane-associated proteins for immune evasion and tissues targeting, NPs could effectively target diseased tissues in the bone marrow in mouse models of MM, where they exerted a significant antitumor effect [60].

#### 3.3 Cardiovascular diseases

**Figure 6:**
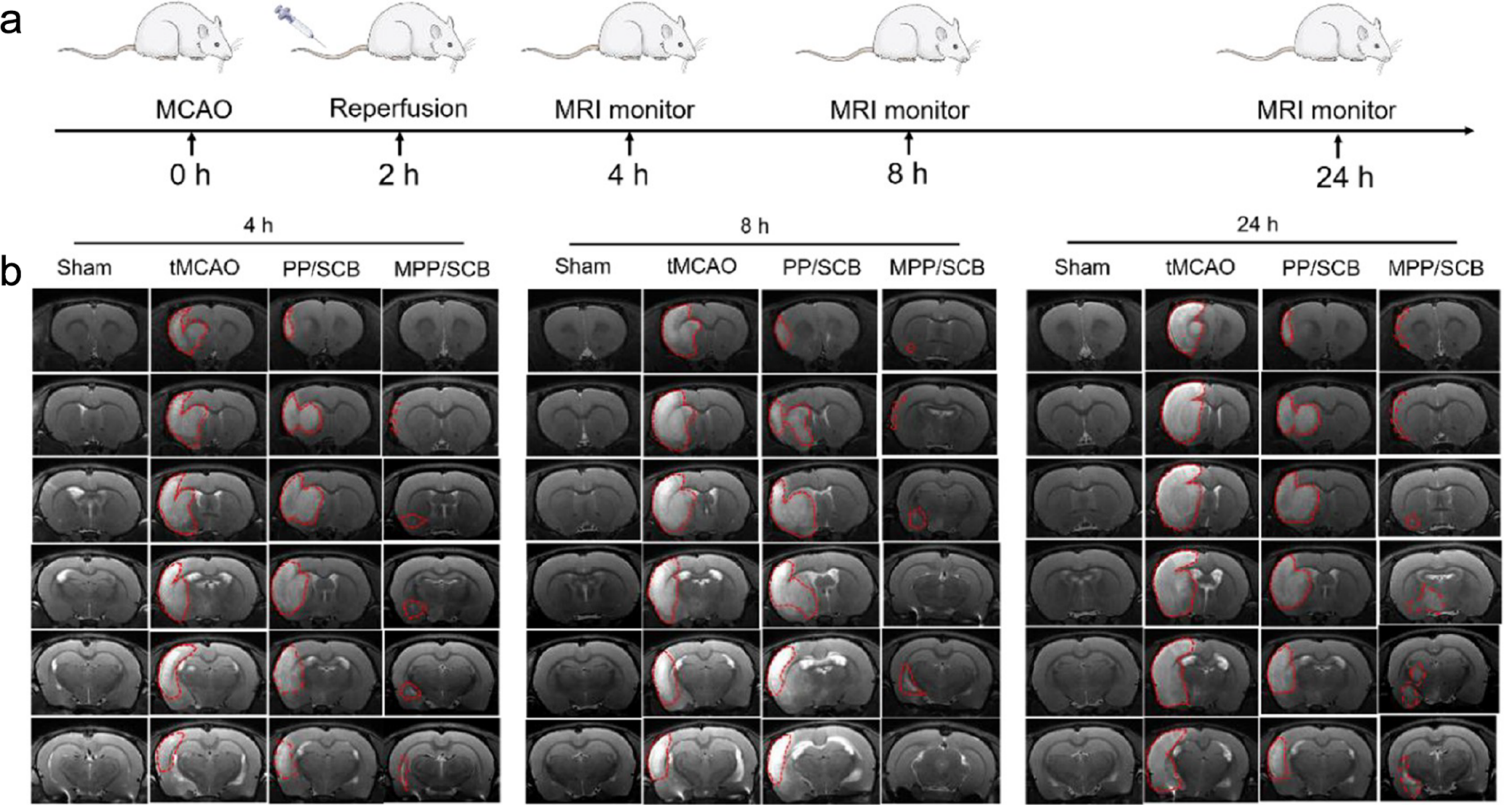
In vivo MRI assay in transient middle cerebral artery occlusion rats. The infarct area of rats treated with 4T1 breast cancer membrane-coated pH-sensitive polymeric nanoparticles loaded with succinobucol (MPP/SCB) was significantly reduced on T2W MRI compared with bare nanoparticles (PP/SCB) and saline (tMCAO). (a) Experimental procedure for rats. (b) MRI visualization of the infarcted regions. Adapted with permission from [26]. Copyright 2021 American Chemical Society. This content is not subject to CC BY 4.0.

Ischemic strokes are mainly caused by atherosclerosis and cardiac embolism and can lead to death or disability [68]. Neuroprotection is an important strategy to reduce neurological deficits caused by ischemia/reperfusion injury, but it is difficult to deliver neuroprotective agents to ischemic lesions [69–70]. Inspired by the brain metastasis of some tumors, He et al. tried to apply the 4T1 breast cancer cell membrane to the treatment of ischemic stroke [26]. Expression of syndecan-1 on breast cancer cell membranes promotes migration across the BBB and increases adhesion to perivascular areas of the brain [50]. The transmembrane glycoprotein VCAM-1 (CD106) on the surface of the breast cancer cell membrane mediates adhesion to leukocytes and the early stages of brain metastasis seeding by combining with integrin VLA-1 (α4β1) [51,71]. Utilizing the preferential accumulation of platelets and leukocytes at the site of brain inflammation, the 4T1 breast cancer cell membrane was coated with NPs loaded with succinobucol (SCB), which protected brain cells from ischemia and reperfusion injury. In transient middle cerebral artery occlusion (tMCAO) rat models, the biomimetic NPs showed preferential accumulation in the ischemic hemisphere while efficiently reducing infarct volume and protecting nerves, resulting in significantly higher efficacy than that of the bare NPs (Figure 6) [26]. The ability of cancer cells to penetrate biological barriers has shown promise for targeted drug delivery in cerebrovascular disease.

#### 3.4 Immune system diseases

Cancer cell membrane-based NPs play a unique therapeutic role in immune system diseases due to their immunomodulatory functions. Systemic lupus erythematosus (SLE) is an incurable chronic autoimmune disease that involves multiorgan damage (e.g., to the skin, joints, kidneys, heart, and brain) [72]. T lymphocytes are considered the key factors in SLE pathogenesis, and the autoantibodies produced by CD4^+^ T cells will destroy tissues [73]. Utilizing the ability of tumor cell membranes to suppress the immune system, cancer cell membrane-coated NPs enable the regulation of the inflammatory microenvironment. Guo et al. used the major histocompatibility complex class I (MHC I)-deficient 4T1 breast cancer cell membrane to coat dexamethasone (DXM)-loaded biomimetic NPs, endowing them with lower immunogenicity, thus, preventing stimulation of the immune system [22]. The NPs enabled the targeted delivery of DXM to inflammatory regions via the cancer cell membrane-expressed adhesion receptor CD44, which inhibits the expression of inflammation-related components [25]. Furthermore, the highly expressed PD-L1 and CD155 on the cancer cell membrane inhibited the co-receptors PD-1 and TIGHT, thereby targeting disease-related CD4 T cells. The NPs exhibited efficient suppression of the inflammatory environment and promoted homeostasis of the immune system, which shows promise for clinical translation.

### 4 Combining current therapeutics with cancer cell membrane-encapsulated NPs

With the ongoing exploration of biomimetic nanomedicine for diagnosis and treatment, various modalities based on cancer cell membranes are emerging. Using immune evasion and homotypic targeting of cancer cell membranes, the drug dosage can be effectively reduced by avoiding elimination by the immune system while achieving higher therapeutic benefits and lower normal tissue toxicity. Different types of applications related to cancer cell membranes are summarized in Table 2 and detailed in the following sections.

**Table 2:** Applications related to biomimetic cancer cell membrane-coated nanotechnology.

Cell membrane types	Core nanoparticle	Drug	Size (nm)	Disease	Application	Ref.

4T1	diselenide-bridged mesoporous organosilica NPs	cisplatin	ca. 80	breast cancer	chemotherapy	[74]
SMMC-7721	folate-functionalized paclitaxel nanocrystals	paclitaxel	230	liver cancer	chemotherapy	[75]
4T1	mesoporous silica NPs	quercetin	115.2	breast cancer	radiotherapy	[76]
HCT-116, MCF-7, HeLa	Pt-integrated hollow polydopamine NPs	apoptin, verteporfin	ca. 250	colon cancer	radiotherapy, X-ray induced PDT^a^	[77]
H22, RAW 264.7	hollow copper sulfide nanoparticles	sorafenib	210	liver cancer	PTT^b^, chemotherapy	[78]
MDA-MB-231	mesoporous silica nanoparticles containing SPIONs^c^	paclitaxel	220	breast cancer	MHT^d^, chemotherapy	[79]
Cal-27	metal-organic framework material PCN-224	chloroquine	150	oral cancer	PDT, autophagy inhibition	[80]
4T1	TPP^e^-decorated nMOF^f^	imiquimod	216.3	breast cancer	SDT^g^, immunotherapy	[81]
B16-F10	Pd-based coordination porphyrin nanozyme	—	74	melanoma	CDT^h^, PDT, SDT	[82]
MDA-MB-231	PVP^i^-dispersed nMOF of Fe-TCPP^j^	tirapazamine	201	breast cancer	ferroptosis, PDT, chemotherapy	[39]
MC38	poly(citrate peptide)	miRNA365 mimic	295	colon cancer	gene therapy	[55]
B16-OVA, DCs^k^	PLGA	—	ca. 135	melanoma, liver cancer, TC-1 tumor	immunotherapy	[46]
HCT116	poloxamer 407	imiquimod	60	colon cancer	tumor vaccines, immunotherapy	[45]
H460	SPIONs	—	158.2	lung cancer	MRI^l^, immunotherapy	[54]
K7M2	hollow MnO_2_ NPs	ginsenoside Rh2, alendronate	141.5	osteosarcoma	MRI, CDT, immunotherapy	[83]
4T1	Hollow mesoporous Prussian blue NPs	ionidamine, ᴅʟ-menthol	250	breast cancer	PA^m^/US^n^/photothermal multimodal imaging, PTT	[84]
MCF-7, DC	Au core/Pt skin composite NPs	—	45.2	breast cancer	cancer cell detection, cancer therapy	[85]

^a^PDT: photodynamic therapy; ^b^PTT: photothermal therapy; ^c^SPIONs: superparamagnetic iron oxides nanoparticles; ^d^MHT: magnetic hyperthermia; ^e^TPP: triphenylphosphonium; ^f^nMOF: nanoscale metal organic framework; ^g^SDT: sonodynamic therapy; ^h^CDT: chemodynamic therapy; ^i^PVP: polyvinyl pyrrolidone; ^j^TCPP: tetrakis(4-carboxyphenyl)porphyrin; ^k^DCs: dendritic cells; ^l^MRI: magnetic resonance imaging; ^m^PA: photoacoustic; ^n^US: ultrasound.

#### 4.1 Traditional oncology treatment

Although various treatments for cancer have emerged, surgical resection, radiotherapy, and chemotherapy remain dominant in clinical treatment [86]. However, traditional approaches suffer from tumor cell insensitivity and resistance to treatment due to genetic instability and heterogeneity [87]. The limited therapeutic efficacy and toxic side effects on normal tissues hinder the treatment of tumors [88]. Cancer cell bionanotechnology provides a new way to overcome the shortcomings of conventional treatments. Platinum-based drugs are the mainstream first-line chemotherapy for clinical tumors [89]. However, numerous unavoidable problems, such as drug resistance and side effects, have limited their clinical application [89–90]. To overcome these obstacles, cisplatin-loaded NPs encapsulated by 4T1 cell membranes were designed for breast cancer treatment and effectively increased the targeted accumulation in the tumor site. The immune evasion of cancer cell membranes prolong the time that drugs spend in the blood circulation [74]. The half-life of biomimetic NPs in mice was 18.4 h, which was significantly higher than that of bare NPs (6.6 h) and free cisplatin (3.7 h). Moreover, the retention of biomimetic NPs in the liver and spleen was notably lower than that of bare NPs [74]. This biomimetic strategy reduces the presence of the drug in normal tissues and clearance from the body, which will facilitate precision therapy and reduce the cytotoxicity of the drug to normal tissues. Similarly, as a commonly used chemotherapeutic drug, paclitaxel also faces many limiting problems in clinical cancer treatment. Its bioavailability and antitumor activity in the antitumor process have also been shown to be enhanced by encapsulation with liver cancer cell membranes [75].

Radiotherapy also plays an important role in tumor therapy. Nevertheless, it has been hindered by the insufficient sensitivity of tumor tissue and serious side effects [91]. Thus, exploring more efficient radiosensitizers is a feasible way to improve the efficacy of radiotherapy. By improving the biocompatibility and tumor targeting ability of radiosensitizers, the concentration of radiosensitizers can be effectively increased in tumor tissues, and the side effects on normal tissues can be significantly reduced [92]. Quercetin (QT), a flavonoid with various functions, such as antitumor activity and radiosensitization, has been loaded into NPs and encapsulated by cancer cell membranes to further study its anticancer effects [76]. After QT was delivered to tumor tissue by the active targeting ability of the membrane, the sensitivity to radiotherapy was effectively improved, and a strong anticancer effect was exerted under X-ray irradiation [76]. Gong et al. designed a pH-responsive multifunctional biomimetic nanoparticle with radiosensitizing activity, which showed better biocompatibility and tumor targeting after coating with a cancer cell membrane [77]. Volume and weight of tumors in mice treated with biomimetic NPs and irradiation were significantly reduced compared to those of mice treated with bare NPs. The results showed that the tumor tissues exhibited a higher rate of apoptosis [77]. In summary, cancer cell membrane-mediated bionanotechnology shows promise for precisely targeted and individualized therapies.

#### 4.2 Hyperthermia

Hyperthermia is a cancer treatment method that can convert external energy (e.g., ultrasound, light, radiofrequency, microwave, and magnetic field energy) into heat and increase the temperature in tumor tissues [93–94]. Cancer cells are more sensitive to heating than normal cells. As a result, apoptosis of cancer cells is greater than that of normal tissue when heated above 40 °C [79,95]. The precisely targeted thermal killing of tumor tissue can help to improve the efficiency of energy utilization and reduce power and time during ablation, which will minimize damage to normal tissue around the tumor [95]. Photothermal therapy (PTT) can generate thermal energy by using near-infrared (NIR) radiation to ablate cells or trigger the release of related therapeutic drugs [94,96]. PTT is characterized by noninvasiveness, deep tissue penetration, and high anticancer efficiency, showing good prospects in clinical treatment [97]. Biomimetic NPs of mesoporous polydopamine nanocarriers have been applied to treat prostate cancer [56]. This nanoagent shows good drug-loading capacity and photosensitivity and can be applied in NIR photothermal conversion. After the autophagy inhibitor chloroquine (CQ) was loaded onto the nanocarrier, it was coated with prostate cancer cell membrane for tumor targeting and hyperthermia [56]. In a therapeutic strategy for HCC, hepatoma cell membranes and macrophage membranes were hybridized to obtain the advantages of different cell membranes [78]. When nanocarriers with photothermal conversion ability were used to carry the anticancer drug sorafenib, efficient drug release and photothermal cell killing were realized [78].

Magnetic hyperthermia (MHT) is another hyperthermia strategy, which generates heat under the excitation of a magnetic field [98]. Magnetic NPs have shown promise in diagnosis and therapeutic effects due to their multiple functions (e.g., magnetic resonance imaging, heat production, magnetic manipulation, and enzyme mimics) [99]. Tumor ablation based on magnetothermy is safe for humans as the energy of the magnetic field is only absorbed by magnetic NPs and not by normal tissue [79]. However, magnetic NPs are prone to aggregation and oxidation, which leads to a decrease in heating efficiency [100]. In addition, magnetic NPs are usually recognized and cleared from the body, which prevents them from reaching their target [101]. Biomimetic cancer cell membrane technology can enhance the performance of magnetic iron oxide NPs (e.g., prevent oxidation, improve biocompatibility, enhance colloidal stability, and enhance targeting), enabling the ablation of tumor tissues by thermal energy [79]. MDA-MB-231 cell membrane-coated NPs loaded with superparamagnetic iron oxide nanoparticles (SPIONs) and PTX were designed for the combination treatment of breast cancer. These biomimetic NPs were able to absorb the energy from a magnetic field for magnetothermal therapy, and synergize with chemotherapy to exert antitumor cell effects [79].

#### 4.3 Reactive oxygen species-related treatments

Emerging noninvasive biocatalytic therapies based on reactive oxygen species (ROS) have attracted widespread attention. Activation of the agent in vivo triggers the production of ROS from intracellular oxygen, which can induce an antitumor response. Due to the engineerable nature of nanotechnology, combining NPs with catalysts and drugs can promote ROS production and effectively improve antitumor efficacy [102]. The coating with cancer cell membranes can confer a more targeted therapy and longer circulation time. Through the enrichment of NPs on tumor tissues mediated by cancer cell membrane proteins, ROS will be induced to exert targeted antitumor effects. Furthermore, it has also been demonstrated that cell membrane coating does not affect the ability to generate ROS during treatment [82].

Tumor growth and progression are accompanied by an abnormal microenvironment (e.g., low pH, local hypoxia, overexpression of glutathione (GSH), and high levels of H_2_O_2_) [39]. GSH is the main antioxidant component in cells and can scavenge excess ROS in cancer cells. ROS-related therapeutic effects are promoted by depleting GSH and catalyzing excess of H_2_O_2_ in the TME [103]. Programmed cell death (e.g., necroptosis, pyroptosis, ferroptosis, and cuprotosis) regulates the development of cells and plays an important regulatory role in the progression of cancer [104–105]. Iron-dependent ferroptosis is a new type of programmed cell death that is different from other modes of action and molecular mechanisms. It is characterized by mitochondrial lipid peroxidation and reduced levels of GSH peroxidases [106]. Based on the conditions in the TME, ferrous/ferric ions can catalyze H_2_O_2_ to produce hydroxyl radicals (•OH) through the Fenton reaction [39,107]. Nanoscale agents with ferroptosis function coated by cancer cell membranes can avoid surveillance of the body and travel to tumor tissues. This effective inhibition of the TME improves antitumor efficiency by depleting GSH and generating ROS [39]. Similarly, chemodynamic therapy (CDT) can utilize the Fenton reaction to convert endogenous H_2_O_2_ into ROS, resulting in a toxic effect on cancer cells [108]. Biomimetic NPs of porphyrin-based nanozymes were designed and successfully delivered to the TME with encapsulation by the melanoma B16F10M cell membrane [82]. They can generate •OH by catalyzing excess H_2_O_2_ to exert a toxic effect on tumor cells [82]. This precise delivery of anticancer modalities shows great significance for enhancing the effects of ferroptosis and CDT.

The utilization of photosensitizers and sonosensitizers in bionanotechnology contributes to the exploration of more efficient tumor treatment strategies. Photodynamic therapy (PDT) can activate photosensitizers by light radiation and induce the production of singlet oxygen and superoxide in vivo [109]. It is important to reduce dosages and systemic toxic effects, which can be achieved by increasing the ability for uptake and for avoiding clearance of photosensitizers in tumor tissue. Due to its shallow location and high clinical operability, oral cancer shows high application potential regarding PTT and PDT [110]. A CQ-loaded nanoformulation based on cancer cell membranes was designed for the treatment of oral squamous cell carcinoma by the Dai group [80]. After high accumulation in tumor tissue, the nanoformulation can be activated under 660 nm laser irradiation and generate ROS, which synergizes with the subsequent release of CQ for anticancer effects. In the early stages of intervention with nanoagents and laser irradiation, both bare NPs and cancer cell membrane-encapsulated NPs exhibited good tumor suppressive effects. However, the tumors in mice treated with bare NPs began to gradually increase in size after day 12 of treatment, whereas the biomimetic NPs still exhibited efficient antitumor effects [80]. In contrast to the poor tissue penetration of PDT, ultrasound-based sonodynamic therapy (SDT) shows stronger tissue penetration [111]. After absorbing energy, the ultrasound sensitizer exerts antitumor efficacy through various pathways, such as ROS generation, mechanical effects, thermal effects, and immune system-oriented efficacy [111–112]. Based on the diverse functions of SDT, Luo et al. synthesized porphyrin-based metal-organic NPs with ultrasound-responsive properties to target mitochondria [81]. Through the camouflage of the cancer cell membrane, the NPs can avoid clearance and target homologous mitochondria [81]. Tumor cells can be killed by the multiple effects of SDT, and the subsequently released tumor-associated antigen further induces antitumor immunity in the body through presentation [81]. This tumor treatment strategy has shown promise due to its noninvasiveness and specific selectivity.

#### 4.4 Gene therapy

Gene therapy can modulate tumorigenesis and progression by delivering transfection-related components to tumor tissue, such as mRNA, siRNA, miRNA, and sgRNA [113]. This strategy can avoid the impact on normal tissues and shows the advantage of low cytotoxicity. However, RNA is highly unstable and can be easily degraded and eliminated by the kidney [114]. Gene delivery technology based on biomimetic NPs shows improved RNA delivery efficiency and overcomes the obstacles of body clearance, insufficient targeting, and low biocompatibility [115]. PLGA NPs loaded with siRNA-E7 and PTX were synthesized for cervical cancer treatment. siRNA-E7 enables the knockdown of E7, which leads to upregulation of the antitumor protein RB and sensitivity to PTX. The biomimetic NPs accumulated three times more than bare NPs in the tumor region after being wrapped by HeLa cell membranes. In subsequent treatments, the biomimetic NPs exhibited an efficient tumor suppression rate of 83.6% with no significant toxic effects on major organs [115]. miRNA365 has been confirmed to inhibit tumor cell development and promote tumor cell apoptosis in the colon and other tumors [116]. Zhang et al. achieved efficient gene transfection in tumor tissue by preparing homologous colon cancer membrane-coated biomimetic NPs [55]. These biomimetic NPs consists of poly(citrate–peptide) (PCP) and miRNA365 mimic as the core and are coated with the MC38 cell membrane. After targeting tumor tissue, the NPs exert antitumor effects by inhibiting the expression of Ki67/Bcl2 and enhancing tumor cell apoptosis [55]. This strategy combining gene therapy with biomimetic technology has been shown to effectively improve the biocompatibility and bioavailability of drugs.

#### 4.5 Immunotherapy

Traditional tumor treatment methods (e.g., surgical intervention, radiotherapy, and chemotherapy) have a certain effect in clinical antitumor treatment and can prolong the survival time of patients. However, they are insufficient in dealing with metastasis and recurrence. The emergence of cancer immunotherapy has changed the traditional tumor treatment model and brought hope to tumor patients, especially those with advanced malignant tumors. Cancer immunotherapy has become a long-term solution for cancer treatment by inducing an antitumor immune response and lasting systemic protection. However, it also faces problems such as a low immune response rate, an inability to effectively predict immune efficacy, and insufficient targeting ability [117]. Various antigens are present on the surface of cancer cell membranes, which can act as tumor-specific antigens and be extracted by antigen presenting cells [45]. The co-incubation of dendritic cells (DCs) with cancer cell membrane-coated NPs enables the processing and presentation of multiple antigens from the source cells [46]. The DC membrane showing tumor-associated antigenic epitopes was used to encapsulate PLGA NPs to prepare the new biomimetic nanoformulations. After entering the blood circulation, it can directly cross-prime T cells to produce a strong antitumor response [46]. Nanovaccine-based immunotherapy can trigger antitumor-specific immune responses and establish long-term immune memory [118]. Cancer cell membranes have become a unique method for the preparation of biomimetic nanovaccines due to the numerous tumor-specific antigens carried on their surfaces [118]. A type of microneedle containing biomimetic NPs with the HCT116 cell membrane was developed for the treatment of colon cancer [45]. Through simple skin delivery and the subsequent presentation of relevant antigens, it can endow the body with efficient antitumor immunity [45]. Moreover, the nanovaccine integrated with individualized antigens extracted from postoperative colon tumor tissue has been demonstrated to exert highly effective tumor suppression in a postoperative tumor model. After synergistic treatment with an immune checkpoint inhibitor, this therapeutic strategy showed a 60% postoperative cure rate for MC38 colon tumors [119].

### 5 Application of biomimetic NPs in theranostics

Cancer cell membranes have also been extensively employed to improve the sensitivity and specificity of diagnostically relevant agents. The combination with biomimetic technology will contribute to the early diagnosis and intervention for patients and has shown promise for medical prospects. Some of the applications related to biomimetic cancer cell membrane-coated agents are listed and described below.

#### 5.1 Magnetic resonance imaging

**Figure 7:**
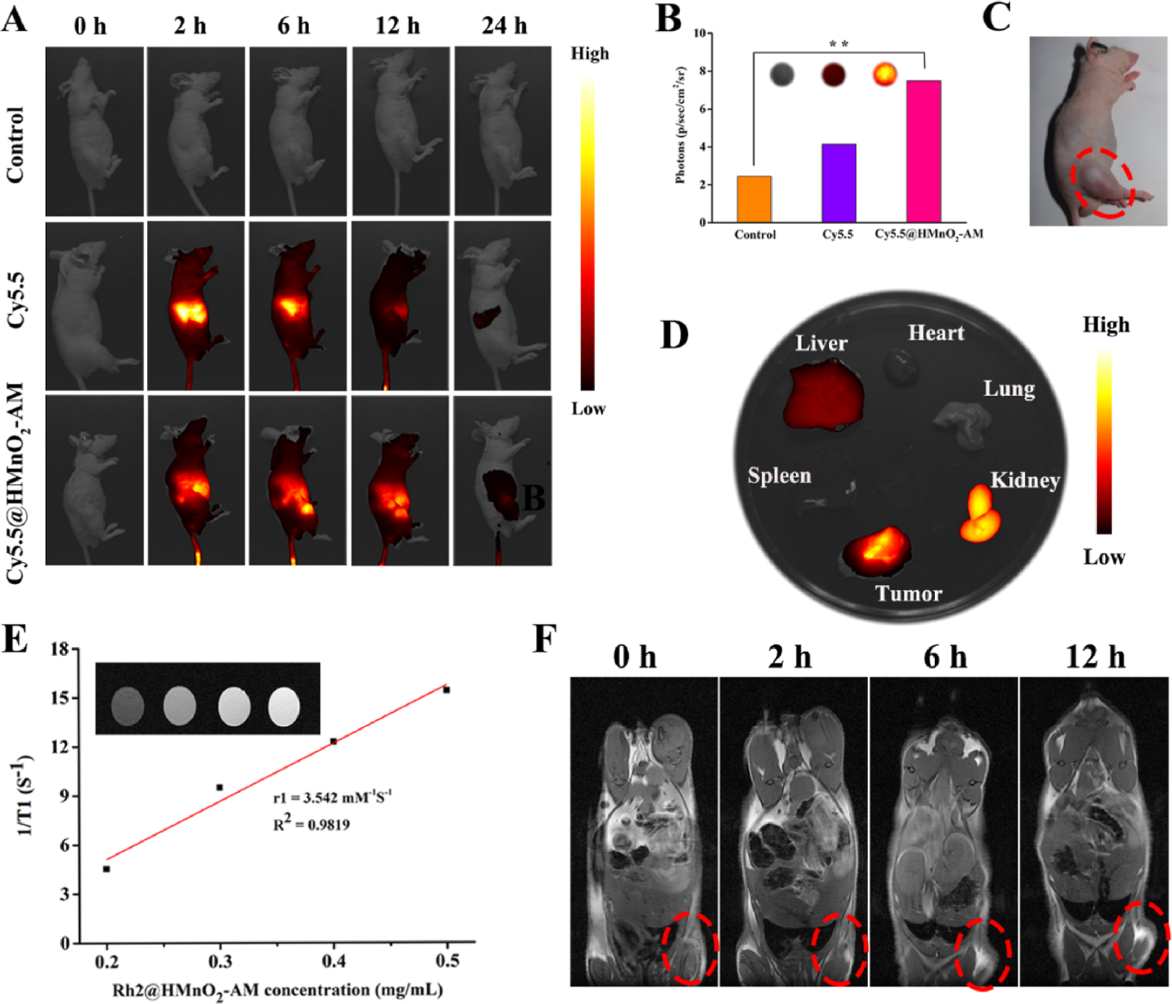
Distribution of K7M2 cell membrane-coated hollow manganese dioxide nanoparticles loaded with alendronate and the fluorescent dye Cy5.5 (Cy5.5@HMnO2-AM) in mice. (A) In vivo fluorescence imaging. (B) Statistical analysis of the fluorescence intensity of tumors in different treatment groups. (C) Mouse image. (D) Ex vivo fluorescence imaging of major organs. (E) T1-weighted MR image of the biomimetic nanoparticles. (F) T1-MRI of mice. Reproduced from [83]. (© 2022 Fu L et al., published by Elsevier, distributed under the terms of the Creative Commons Attribution-NonCommercial-NoDerivs 4.0 International License, http://creativecommons.org/licenses/by-nc-nd/4.0/). This content is not subject to CC BY 4.0.

Magnetic nanoparticles are widely used in magnetic resonance imaging (MRI) because they can improve imaging by enhancing proton relaxation in tissues [120]. Among them, superparamagnetic iron oxides (SPIONs) are widely used with numerous advantages, such as small size, colloidal stability, low toxicity, magnetic heating properties, and enhanced molecular MRI [121]. However, SPIONs cannot be effectively distributed in tumor tissue for effective imaging because of the lack of targeting capability. An appropriate surface coating could enhance their diagnostic value for medical imaging [122]. Biomimetic nanodelivery systems are of great significance for imaging agents [101]. H460 lung cancer cell membrane-coated NPs carrying SPIONs have been engineered for the diagnosis and treatment of lung cancer and enabled MRI imaging of tumor tissue in mouse lung cancer tumor models. Moreover, this nanodelivery system also yielded controlled release in the TME and exerted a tumor-inhibitory effect through the incorporated surface-modified PD-L1 inhibitory peptide and MMP2 substrate peptide [54]. Manganese oxide (MnO_2_)-based NPs function as MRI imaging agents and can also utilize Fenton-like reactions to deplete GSH and generate •OH to mediate tumor cell death [123]. In the work of Fu et al., a hollow MnO_2_ NP-based nanocarrier was encapsulated with a cancer cell membrane, which endowed the NPs with the ability to target tumor tissues and mediate tumor killing through chemical kinetics [83]. In addition, further anticancer effects can be exerted by the ginsenoside Rh2, which was delivered by nanocarriers and inhibited the growth of cancer cells and induced tumor apoptosis. The accumulation of the fluorescent signal of NPs in tumor tissues reached the highest value 8 h after the injection of the biomimetic nanoformulations (Figure 7A–D). The biomimetic NPs showed good contrast in T1-weighted imaging after high accumulation in the tumor tissue (Figure 7E,F) [83].

#### 5.2 Photoacoustic imaging

Ultrasonography is a common noninvasive clinical real-time imaging technique of with low cost, and ease of operation. Based on the photoacoustic (PA) effect, a hybrid imaging modality of PA imaging has been derived, which has shown advantages in better tissue penetration and spatial resolution [124]. Since US and PA commonly depend on the signal conversion of US transducers, the combination of US and PA dual-mode imaging shows good integration and enables the high-quality real-time imaging of tumors [125]. A biomimetic nanoagent with the ability to target tumor tissue and enable multimodal imaging was fabricated for more efficient diagnosis and treatment by the Shu group. The biomimetic nanoformulation utilized hollow mesoporous Prussian blue nanoparticles as the core, endowing it with photothermal conversion and photoacoustic imaging capabilities. The loading of lonidamine, which has anticancer function, and ᴅʟ-menthol, which exhibits controlled release, enabled the NPs to function more effectively in diagnosis and treatment. After encapsulation by the 4T1 breast cancer cell membrane, the NPs had good stability and were effectively transported to the tumor tissue. This strategy yielded precise photothermal killing of cancer cells and enabled US/PA imaging of the tumor tissue. In addition, the local temperature rise of the tumor tissue can be displayed on the infrared camera, which helps to monitor the treatment temperature in real time [84].

## Conclusion

Biomimetic cancer cell membrane-coated NPs present unique advantages that provide new breakthrough ideas for various obstacles in the field of biomedical treatment. Numerous biomimetic nanoagents based on cancer cell membrane-related functions have been reported and demonstrated strong clinical translation potential. In this review, the application in different fields of diseases and the existing diagnosis and treatment methods have been described in detail. In the face of malignant neoplasms, the homotypic cell membrane yields excellent functions (e.g., homologous targeting function, immune evasion ability, and overcoming various biological barriers) to deliver the formulation to the target site for further treatment. Furthermore, tumor vaccines based on the surface antigens of the cancer cell membrane are expected to be a powerful method against tumors, which can also prevent tumor recurrence by establishing long-acting antitumor memory. In nontumor diseases, cancer cell membranes are also a unique therapeutic approach in cerebral ischemic lesions and autoimmune diseases because of their chemotactic ability to target ischemic lesions and their strong immunosuppressive function. The many functions of cancer cell membranes result from multiple complex protein interactions, which are more powerful than traditional single-component modifications. Various emerging approaches and improvements to conventional treatment methods have been explored and applied to clinically relevant diagnoses. However, the nature of foreign substances and the inability to efficiently concentrate drugs on specific tissues are unavoidable problems during treatment that lead to unnecessary waste of pharmaceuticals, strong toxic side effects in the body, and failure to achieve the expected therapeutic benefits. The biomimetic cancer cell membrane technology shows good prospects for individualized treatment. This facilitates the reduction of drug doses and efficient diagnosis and treatment.

Although NPs based on cancer cell membranes have been found to be a promising biomimetic technology, many challenges still need to be addressed for further application in medical diagnosis and therapy. Biomimetic nanotechnology using cancer cell membranes is still in its infancy. The mechanisms of the intricate functions of numerous types of proteins on the surface of cancer cell membranes need to be further researched. In addition, there is a considerable lack of research on potential side effects and long-term systemic toxicity in humans. Despite promising clinical translation prospects, diagnostic and therapeutic experimentation with biomimetic cancer cell membrane-coated NPs are still in the animal experiment stage. The construction of preclinical models that closely mimic the human environment is helpful for the development of clinical diagnosis and treatment. Furthermore, the technology for preparing biomimetic cancer cell membrane-coated nanoagents is not yet mature, and various constraints in the preparation process (e.g., production cost and preparation purity) need to be addressed and overcome. In summary, biomimetic cancer cell membrane nanotechnology has opened up a new area of clinical diagnosis and treatment, showing good potential for future clinical translation.
